# Targeted Interneuron Ablation in the Mouse Hippocampus Can Cause Spontaneous Recurrent Seizures

**DOI:** 10.1523/ENEURO.0130-17.2017

**Published:** 2017-07-21

**Authors:** Jay Spampanato, F. Edward Dudek

**Affiliations:** Department of Neurosurgery, University of Utah School of Medicine, Salt Lake City, UT 84108

**Keywords:** CA1 ablation, epilepsy, GABAergic, local field potential, status epilepticus

## Abstract

The death of GABAergic interneurons has long been hypothesized to contribute to acquired epilepsy. These experiments tested the hypothesis that focal interneuron lesions cause acute seizures [i.e., status epilepticus (SE)] and/or chronic epilepsy [i.e., persistent spontaneous recurrent seizures (SRSs)]. To selectively ablate interneurons, *Gad2-ires-Cre* mice were injected unilaterally in the CA1 area of the dorsal hippocampus with an adeno-associated virus containing the diphtheria toxin receptor (DTR). Simultaneously, an electrode, connected to a miniature telemetry device, was positioned at the injection site for chronic recordings of local field potentials (LFPs). Two weeks after virus transfection, intraperitoneal injection of DT consistently caused focal, specific, and extensive ablation of interneurons. Long-term, continuous monitoring revealed that all mice with DT-induced interneuron lesions had SRSs. Seizures lasted tens of seconds and interseizure intervals were several hours (or days); therefore, these interneuron lesions did not induce SE. The SRSs occurred 3-5 d after DT treatment, which is the estimated time required for DT-induced cell death; therefore, induction of SRSs occurred without the latent period typical of acquired epilepsy. In five of six DT-treated mice, SRSs stopped within days, suggesting that the DT-induced interneuron lesions did not usually cause epilepsy. In one mouse, however, SRSs occurred for ≥34 d after interneuron ablation, similar to epilepsy after experimental SE. Sham control mice had no detectable seizures, confirming that the SRSs were due to ablation of interneurons. These data show that selective interneuron ablation consistently caused SRSs but not SE; and, at least under the conditions used here, interneuron lesions rarely led to persistent SRSs (i.e., epilepsy).

## Significance Statement

Cell death, including interneuron loss, is a common pathology of the brains of human patients and animal models of acquired epilepsy. However, the degree to which interneuron loss is a cause of or is a result of seizures remains unknown. By specifically ablating interneurons of the dorsal hippocampus, the degree to which selective interneuron lesions could cause spontaneous seizures was examined with long-term, continuous recordings of behavior (i.e., with video) and local field potentials (LFPs). Interneuron loss consistently caused seizures, which (1) were separated by intervals too long to be status epilepticus (SE) and (2) usually stopped after only a few days, and thus did not appear equivalent to epilepsy.

## Introduction

The hypothesis that reduced synaptic inhibition can lead to seizures began with the discovery that penicillin, which was known to cause seizures when applied to the cortex, is a GABA_A_-receptor antagonist (Curtis et al., 1972; van et al., 1973; Ebersole and Levine, 1975; Anderson and Rutledge, 1979). The determination that GABAergic neurons and their synapses were lost in and around epileptic foci led to the hypothesis that interneuron death or dysfunction could cause epilepsy (Ribak et al., 1979; Houser et al., 1986; Ribak et al., 1986; Roberts, 1986). Research on animal models of epilepsy and tissue from human patients with temporal lobe epilepsy (hTLE), both postmortem and from surgical resections, has revealed interneuron loss in both focal and generalized epilepsies (Cossart et al., 2005; Maglóczky and Freund, 2005), thus further supporting this possibility.

The loss of interneurons in animal models and hTLE is complicated, unresolved and controversial: no interneuron group is lost entirely and many classes of interneurons experience some degeneration depending on the region of the brain and the type of precipitating insult (Huusko et al., 2015). Although interneuron loss is well-documented, the extent to which this change is a cause of spontaneous recurrent seizures (SRSs), rather than an effect of the seizures themselves, remains unknown. This is partly because the animal models of TLE generally involve an induction period of repetitive activity and/or seizures [e.g., status epilepticus (SE)], which has multiple and widespread effects. This initial insult is typically followed by a latent period, during which interneuron loss can be observed before the establishment of SRSs (Dinocourt et al., 2003). These studies suggest that interneuron loss is a factor in the establishment of an epileptic network, but not necessarily a cause of seizures or epilepsy by itself. Previous studies have largely been correlative, and a direct test of this hypothesis has previously been difficult, if not impossible.

We have used recent advances in mouse genetics combined with targeted viral transfection to directly ablate a discreet nonspecific population of interneurons in the CA1 subregion of the hippocampus. We hypothesized three possible outcomes of this manipulation. (1) If the insult was too small, the anticipated reduction in inhibition would not cause seizures. (2) If the insult was too large, the resulting imbalance of excitation and inhibition could lead to SE, which could kill the mice unless the seizures were blocked pharmacologically. (3) If the insult was sufficient to cause SRSs, the seizures could be persistent and could possibly increase in severity and frequency, as is seen in the progressive course typical of acquired epilepsy. The data described in the present manuscript show that interneuron ablation in the dorsal CA1 area consistently caused SRSs. Thus, as expected, interneuron loss alone in the neural network of an otherwise normal laboratory mouse appears to be sufficient to consistently cause SRSs. The interval between seizures was >30 min in duration and thus too long to be representative of SE, despite the relatively large size of the interneuron lesion. The pattern of SRSs was similar to epilepsy; however, in five of six mice, the SRSs only occurred for a few days, which did not meet the definition of chronic epilepsy. The SRSs persisted for approximately one month in one mouse, suggesting that focal interneuron ablation, at least under some conditions, can by itself lead to chronic epilepsy.

## Materials and Methods

### Mice

*Gad2-ires-Cre* mice (Taniguchi et al., 2011) were originally purchased from The Jackson Laboratory (stock number 010802) and were subsequently maintained on a C57/Bl6 background through homozygous breeding. All procedures were performed with protocols approved by the University of Utah Animal Care and Use Committee and in accordance with NIH guidelines for the care and use of laboratory animals. After surgical procedures, mice were housed individually with a 12/12 h light/dark cycle and with food and water *ad libitum*. Similar aged male and female mice were used for each separate set of experiments in this study. For the *ex vivo* electrophysiology, three littermates were selected for each set of experiments. Ages ranged from 58-89 d at time of the whole-cell recordings (control mice average age, 78 d; experimental mice average age, 76 d). For immunohistochemistry, two sets of littermates were used across groups and those mice were 94 d old at time of perfusion. For chronic monitoring, mice were 69-111 d old at the time of the transfection/implant (control mice average age, 95 d; experimental mice average age, 84 d).

### *In vivo* ablation

The *FLEX-DTR-GFP* construct (Azim et al., 2014; Fink et al., 2014) was used to generate *AAV2-FLEX-DTR-GFP* viral particles (AAV-DTR) by the Penn Vector Core at The University of Pennsylvania. In brief, the viral construct encodes for the Cre-dependent expression of simian *DTR* (heparin-binding EGF-like growth factor) fused to *GFP* (Buch et al., 2005). The fusion *DTR-GFP* is floxed by two pairs of heterotypic, antiparallel *loxP* recombination sites so that Cre-mediated recombination reverses the backwards orientation of the construct. Virus was purchased as a small scale custom preparation with a guarantee yield of 0.5-1 ml volume of 2 × 10^12^ genome copies.

A 1-μl volume of AAV-DTR was targeted to the dorsal CA1 area of the hippocampus via stereotaxic injection using the coordinates from bregma: anterior-posterior (AP), -1.85; medial-lateral (ML), -1.25; dorsal-ventral (DV), -1.05. Mice were anaesthetized with 4% isoflurane (MWI Veterinary Supply), maintained at >1% during the procedure, and positioned in a head-fixed stereotaxic frame. Equipment was kept sterile throughout the procedure with 70% ethanol and a heated glass bead sterilizer. Hair was removed from the scalp, and the incision site was prepped with alcohol and betadine. A 0.25-inch incision was made down the midline of the animal’s head to expose the skull. After the incision was made, the skin was pulled aside and clamped with aneurysm clips to ensure access to the surgical field on the top of the skull. The periosteum was removed using sterile cotton swabs dipped in 0.3% hydrogen peroxide. A small burr hole was drilled into the skull using a circuit board drill (CircuitMedic) above the dorsal hippocampus (using the above coordinates) to expose the cortex. An infusion cannula was lowered into position and a steady rate (15 μl/h or 0.25 μl/min) injection was controlled by a single-syringe infusion pump (Cole-Parmer). At the completion of the injection, the cannula was left in place for 10 min before removal. The scalp was sutured and the mice were allowed to recover before returning to the home cage. At two to three weeks after transfection, diphtheria toxin (DT; 30 ng/g, Sigma-Aldrich) was administered once daily for 2 d via intraperitoneal injection. Virus controls received viral transfection and intraperitoneal injection of saline. Toxin controls received saline transfection and intraperitoneal injection of DT. Several preliminary experiments were conducted to optimize the targeting and expression of the virus. When volumes larger than 1 μl were used, significant spread was observed within the hippocampus beyond CA1, to the ascending cortex, as well as across the midline into the contralateral hippocampus. Smaller volumes resulted in incomplete fill of the CA1 region. No ventricular targeting was observed with the coordinates reported above.

#### Immunohistochemistry

**Figure 1.**
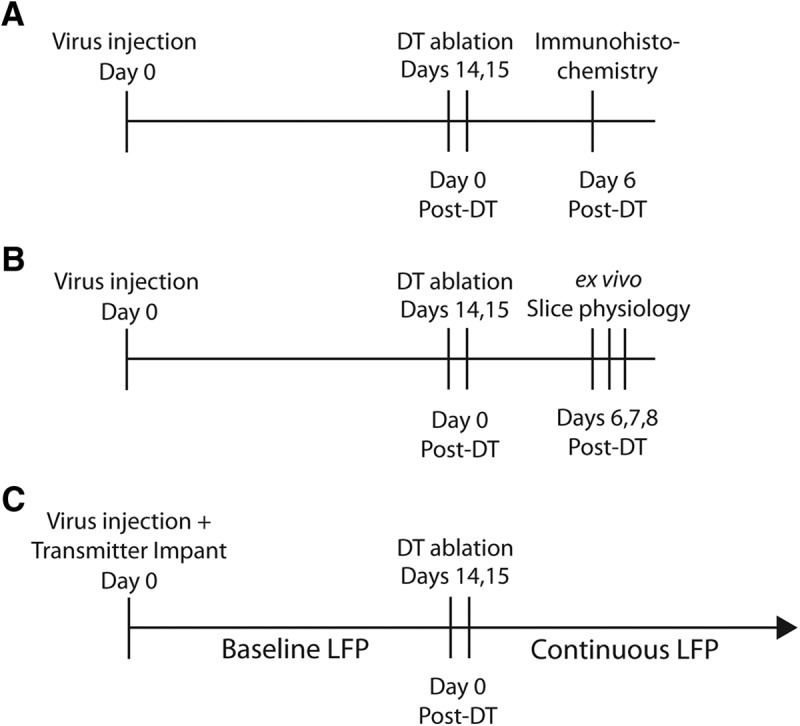
Time line of the experimental protocols. ***A***, For immunohistochemical analysis of the efficacy of DT-induced interneuron ablation, mice were transfected with virus on day 0, followed by DT treatment on days 14 and 15. Six days after the first DT injection, mice were perfused and tissue was harvested and processed for antibody labeling of GFP and GAD67. In a separate set of experiments, virus expression was confirmed at day 14 after transfection. ***B***, To determine the functional result of interneuron ablation, a similar timeline was followed with the exception that on day 0, three littermates were transfected with virus and then on each of the days 6-8, one of those three mice was used for *ex vivo* slice recordings. This experiment was repeated at least three times with the animal treatment condition (experimental or controls) rotated through days 6-8. This series of experiments was also conducted with *ex vivo* recordings performed on days 2-4. ***C***, To determine the effect of interneuron ablation on whole brain function *in vivo*, mice were implanted with a wireless transmitter at the time of virus transfection (day 0 of the experiment and day 14 relative to DT treatment). A 14-d baseline LFP was recorded before induction of ablation, followed by continuous LFP recording.

At 6 d after DT (Fig. 1*A*), mice (*n* = 9, three experimental and three of each control) were euthanized with an overdose of sodium pentobarbital (100 mg/kg, i.p.). Anaesthetized mice were transcardially perfused with saline (0.9% NaCl) followed by 4% paraformaldehyde (PFA) in 0.1 M phosphate buffer (PB; pH 7.2). Brains were removed and postfixed in the same fixative by immersion for ∼2 h at room temperature on an orbital shaker, then 4°C overnight. The following day, brains were washed in 0.1 M PBS (pH 7.2). Coronal sections 50 µm thick were cut using a Leica VT-1000S Vibratome (Leica Microsystems) and collected serially into a multiwell chamber. Sections were blocked in 5% BSA, 0.1% Triton X-100, and 0.05% sodium azide in 0.1 M PBS for 1 h, and then washed in PBS. Primary antibodies: GFP (1:2000, rabbit polyclonal AB3080P, EMD Millipore) and glutamic acid decarboxylase 67 (GAD67; 1:10000, mouse monoclonal MAB5406, EMD Millipore) were diluted in 0.1 M PBS containing 0.5% BSA, and 0.05% sodium azide, and incubated on an orbital shaker for >48 h at room temperature. Sections were washed in PBS and then incubated for >5 h in biotinylated anti-mouse IgG (1:500, Vector Laboratories) diluted in 0.1 M PBS containing 0.5% BSA, and 0.05% sodium azide. Sections were washed again, and then incubated overnight in streptavidin conjugated Alexa Fluor 555 (1:2000, Life Technologies) and anti-rabbit IgG Alexa Fluor 488 (1:2000, AP132JA4, EMD Millipore) overnight. Sections were washed in PBS and then normal saline and mounted in Fluoromount-G (SouthernBiotech) for fluorescence microscopy.

For cell counting, serial sections (as described above in this section) were collected through the dorsal hippocampus into three wells of a six-well tissue culture plate beginning with approximately bregma -0.9 and ending when each well contained 14 sections (42 sections total). Following the staining procedure, sections from each well were then mounted on to individual slides in three rows of four sections each in anatomically sequential order beginning with the most rostral tip of the hippocampus using hippocampal morphology and other brain regions as landmarks for proper ordering. GAD67-labeled cells were counted from one slide (every third section) starting with the section at approximately bregma -1.34 and ending after eight counted sequential sections. The CA1 boundary was defined dorsal by the cortical fiber tract, ventral by the hippocampal fissure, medial by the midline and lateral by the transition to CA2. Counting was performed on a Zeiss Imager.Z1 (Zeiss) using a 20× objective (0.8 NA) focused on the surface plane of the tissue. Only cells that were in focus in this plane were counted due to incomplete penetrance of the antibody (GAD67) through the tissue. Cells were defined as a mostly complete cell body including a visible nucleus. No cell fragments were included in the counts. Cell counts were performed blind to treatment and to maintain the blind, GFP-labeled cells were counted only after the GAD67 cell counts were finalized.

#### Electrophysiology

On days 2-4 and days 6-8 after DT treatment (Fig. 1*B*), mice were anesthetized with isoflurane and decapitated. Brains were removed rapidly and transferred to ice-cold choline chloride artificial CSF (aCSF) containing: 118 mM C_5_H_14_ClNO, 2.5 mM KCl, 2.5 mM CaCl_2_, 10 mM MgCl_2_, 1.2 mM NaH_2_PO_4_, 10 mM glucose, 3 mM kynurenic acid, and 25 mM NaHCO_3_, bubbled with carbogen (95% O_2_, 5% CO_2_) to yield pH 7.3-7.4. Coronal slices (300 μm) containing the dorsal CA1 region of the hippocampus were cut using a Leica VT-1000S Vibratome (Leica Microsystems), and transferred to normal aCSF: 125 mM NaCl, 3 mM KCl, 1.3 mM CaCl_2_, 1.3 mM MgSO_4_, 1.2 mM NaH_2_PO_4_, 25 mM glucose, and 25 mM NaHCO_3_, and bubbled at 32-34°C. Slices were incubated for at least 1 h before experimentation.

Individual slices were transferred to a recording chamber, submerged, and perfused (∼3 ml/min) with carbogen-gassed recording solution (normal aCSF + 1 μM tetrodotoxin) maintained at 34 ± 2°C using an inline heater. Microelectrodes (3–6 MΩ) were pulled from borosilicate glass and filled with a solution containing: 135 mM Cs methanesulfonate, 8 mM NaCl, 10 mM HEPES, 2 mM Mg_2_-ATP, 0.3 mM Na_3_-GTP, 0.5 mM EGTA, 7 mM phosphocreatine, and 8 mM biocytin; pH 7.3, ∼310 mOsmol. Whole-cell recordings were performed on visually identified pyramidal cells in the dorsal CA1 area using an upright microscope with infrared-differential interference contrast optics (Nikon Eclipse E600FN, Nikon). Data were collected using ClampEx (Molecular Devices) and a Multiclamp 700B (Molecular Devices). Signals were filtered at 3 kHz and digitized at 20 kHz using a Digidata 1322A (Molecular Devices).

Whole-cell recordings of miniature EPSCs (mEPSCs) were obtained by voltage clamping the cell at the reversal potential for miniature IPSCs (mIPSCs), -70 mV (with series resistance compensation) and mIPSCs were obtained by depolarizing the cell to +10 mV, the approximate reversal potential for mEPSCs. Currents were recorded for 6 min at each potential and series resistance was monitored throughout. One cell was recorded from each of the four sections through the ablation zone (first four sections of the dCA1). Each cell was filled with biocytin and each section was fixed in 4% PFA after the recording. Sections were incubated overnight in PFA and washed in PBS and transferred to blocking solution (as above). Cells were visualized with the fluorescent strep-555 (as above).

Synaptic events were detected using the ClampFit template matching function. Amplitude and interevent interval were calculated on the entire data set including all detected events. Decay kinetics were determined by first compiling 40-60 individual (nonoverlapping) events from the last 2 min of each recording period. The composite average of each period was then fit with a standard double exponential equation:

$$f(x)=A1*e^{\left(-t/\tau 1\right)}+A2*e^{\left(-t/\tau 2\right)}$$

where A is the amplitude of the current that decayed with the corresponding τ. The weighted tau (τ_w_) was calculated from the following equation:

$$\tau_{w}=[A1/(A1+A2)]*\tau 1+[A2/(A1+A2)]*\tau 2$$

Intrinsic properties of input resistance and membrane time constant (τ_w_) were calculated from the double exponential fit of the current decay following a 5-mV, depolarizing, square-pulse from the holding potential of -70 mV. Whole-cell capacitance was read from the capacitance neutralization function of the software control of the voltage clamp.

Statistics were calculated using GraphPad Prism 5 software (GraphPad Software).

### Local field potential (LFP) monitoring

Implantation of the LFP monitoring devices (manufactured by Epitel and available as Epoch from Epitel) began immediately after extraction of the virus transfection cannula (Fig. 1*C*). The transmitters and surgical procedures are similarly described by Zayachkivsky et al. (2013). For recording of LFPs, the implanted electrode was a polyamide-coated stainless steel wire. The recording electrode was sterilized with 70% ethanol, allowed to dry, and positioned over the burr hole used for virus transfection. The implant was slowly lowered into position at: AP, -1.85; ML, -1.25; DV, -1.05. The transmitter was then attached to the skull using cyanoacrylate gel (Loctite 454, Loctite) with accelerator (Loctite 7452, Loctite). Additional cyanoacrylate was applied around the unit and the exposed areas of the skull to stabilize the implant. The area was rinsed with saline, and the skin was sutured with Vicryl 4-0-coated polyglactin 910 sutures (Ethicon). Mice were allowed to recover before housing in a new, clean home cage. Implanted mice were individually housed and racked in the LFP recording set-up immediately after recovery from surgery. LFP recording began within a few days after electrode implantation.

Multiple simultaneous LFP signals (low-pass filtered at 100 Hz by the transmitter) were recorded and digitized by an analog-to-digital converter (MP150; BIOPAC Systems), sampled at 500 Hz, and stored on a computer using AcqKnowledge 4.1.1 software (BIOPAC Systems). Video was recorded using infrared cameras and D-Link D-View (D-Link Corporation/D-Link Systems) or Video Insight Monitor Station (Video Insight) software and stored on 2 TB hard drives (Western Digital).

In all, six control mice and six experimental interneuron-ablated mice were used in this study. The six control mice included four virus controls (mice that received virus and intraperitoneal injection of saline instead of DT) and two toxin controls (mice that received a dorsal CA1 injection of saline and intraperitoneal injection of DT). The recordings from interneuron-ablated mice lasted an average of 35 d after treatment (range, 17-73 d) while the control-animal recordings were maintained for a mean of 61 d after treatment (range, 29-85 d). The recordings included infrequent “drop-outs” where the LFP signal was interrupted. These brief periods were mostly limited to cage changes and specific, identifiable behaviors, such as climbing on the wire food rack of the cage. Throughout the recording periods, we experienced occasional software and/or hardware failures that interrupted both video and LFP. In total, 1401 h of EGG were lost during the 17,808 h of recorded LFP activity (∼8% of the total hours recorded). Each recording continued until the battery of the device died, with the exception of one mouse that died prematurely. At the termination of each recording, animals were perfused for confirmation of the position of the electrode.

Seizures were first identified by blinded manual scanning of the LFP, followed by review of the video at the corresponding time to determine if a putative seizure was convulsive or nonconvulsive (when video was missing no determination was made). LFP data were scanned blind to the treatment that each mouse received, and seizures were initially defined as periods of high amplitude and high synchrony with a distinct evolution in the pattern of repetitive activity, including successive periods of oscillations at different frequencies, and were often followed by a period of baseline suppression (i.e., postictal depression) of the LFP recording before recovery back to a normal baseline (see Results). These events were easy to differentiate from artifacts generated by scratching or grooming that produced prolonged periods of high-amplitude highly synchronized LFP activity, but did not contain a clear pattern of repetitive discharges with distinct periods of oscillations at different frequencies, and were not followed by baseline suppression. Grooming/scratching artifacts were then readily dismissed after a brief training period that included identifying them first in the LFP signal and then confirming the behavior on video. Seizure behavior was confirmed by video and then ranked on a scale that reflected the observed behaviors as follows. (1) No obvious behavior observed. (2) Behavior pauses (if moving), or woke up, also associated with “looking back and forth” (Video 1; at ∼54 s). (3) Period of forelimb clonus with or without head twitching (Video 2; at ∼1 m, 10 s). (4) Forelimb clonus and/or head twitching accompanied by rearing. (5) A combination of (3) and (4) including a loss of posture (Video 3).

Movie 1. Nonconvulsive seizure with behavioral pause associated with “looking back and forth” (at 54 s). 10.1523/ENEURO.0130-17.2017.Movie.1

Movie 2. Convulsive seizure with a period of forelimb clonus. 10.1523/ENEURO.0130-17.2017.Movie.2

Movie 3. Convulsive seizure with forelimb clonus and/or head twitching accompanied by rearing. 10.1523/ENEURO.0130-17.2017.Movie.3

## Results

### Evidence for virus expression

**Figure 2.**
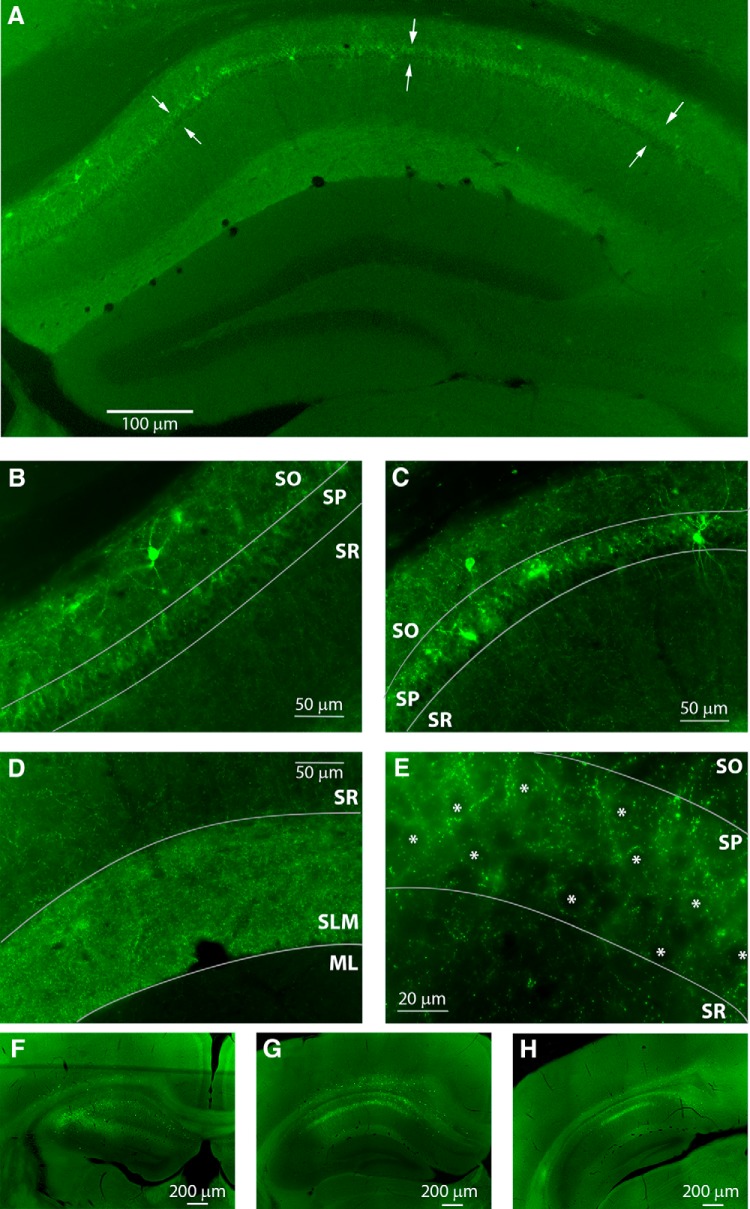
AAV-DTR transfection and expression in the CA1 subregion of the hippocampus. ***A***, Expression of DTR-EGFP can be seen by immunohistochemical labeling with anti-EGFP antibody at two weeks posttransfection. The transfected area extends throughout the medial-lateral expanse of the CA1 region of the hippocampus. Arrows indicate the pyramidal cell body layer of CA1. ***B***, ***C***, Higher power images demonstrate cell-specific expression in the oriens and pyramidal cell layers. ***D***, ***E***, Putative synaptic densities can be seen as puncta in the bright fluorescent band of the SLM region, as well as puncta in the pyramidal cell layer surrounding putative principal cell bodies (asterisks). ***F-H***, Representative images from serial sections from one mouse demonstrate the area of transfection of the dorsal-medial region of CA1. Some expression can be seen in the deep cortical layers. Images were taken from sections corresponding to bregma -1.34 (***F***), -2.18 (***G***), and -2.80 (***H***). The injection was targeted to bregma -1.85.

Viral transgene expression was confirmed via immunohistochemical labeling for GFP. Figure 2 demonstrates virus expression at two weeks after transfection. Within the hippocampus, we observed a consistent confinement of the transfected cells to the CA1 subregion, typically spreading from the ventricle to the CA2/CA3 border in the medial-lateral plane (Fig. 2*A*). A relatively higher number of transfected cells was observed in the stratum oriens (SO) and stratum pyramidale (SP) regions compared to the relatively lower viral transfection in stratum radiatum (SR) and stratum lacunosum-moleculare (SLM). Higher power images (Fig. 2*B*,*C*) illustrate labeling and cellular morphology in SO and SP as well as putative synaptic labeling (puncta) in SLM and SP (Fig. 2*D*,*E*). Serial sections through the hippocampus of one mouse demonstrate the extent of the spread of the virus within the hippocampus and surrounding areas (Fig. 2*F-H*). Together, these data suggest that the cohort of transfected interneurons included the distal dendrite-targeting, oriens lacunosum-moleculare cells as well as the perisomatic-targeting, basket cells (Klausberger and Somogyi, 2008) and is consistent with the extensive and thorough characterization of the specific cre-mediated GFP expression in this model (Taniguchi et al., 2011). Taniguchi et al. (2011) previously demonstrated that GFP expression is restricted to and overlaps with expression of GAD67 (92% specificity and 91% efficiency) and this includes all of the major subpopulations of interneurons (parvalbumin-, somatostatin-, calretinin-, and vasoactive intestinal protein-expressing subpopulations).

### Immunohistochemical evidence for interneuron ablation

**Figure 3.**
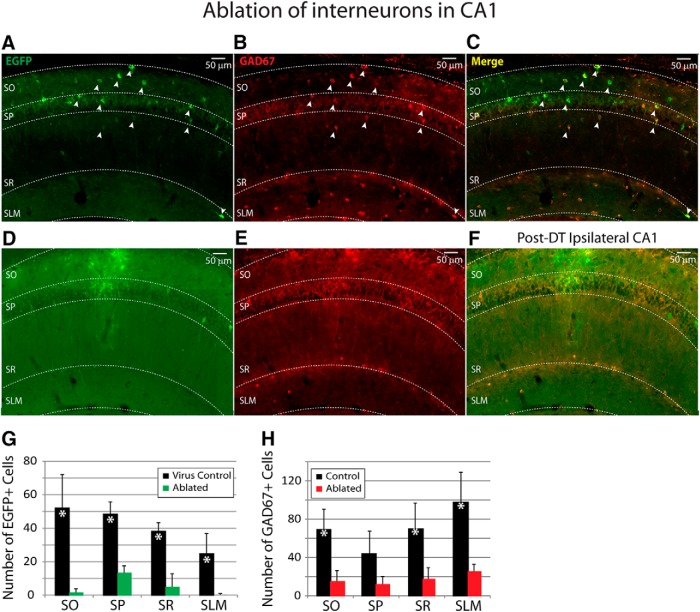
Hippocampal interneurons are lost after targeted ablation by systemic DT administration. ***A-C***, EGFP-expressing interneurons in the CA1 region coexpress GAD67 (arrows). ***D-F***, At 6 d after DT treatment, a significant loss of EGFP-expressing (green, ***D***) and GAD67-expressing (red, ***E***) cells can be seen in the merged image (***F***), across all subregions of CA1. **G.** Quantification of the average number of virus-transfected cells (*n* = 3 mice/group) demonstrated a large population of EGFP-expressing cells in virus-transfected control mice that had not been given DT, and a significant reduction in EGFP-expressing cells in virus-transfected mice that had been given DT. ***H***, Quantification of GAD67-expressing cells (in the same tissue as ***G*** for virus-transfected control and virus-transfected ablated) confirmed that the ablation resulted in a significant reduction of interneurons across all regions in the ablated (*n* = 3) compared to the control mice (grouped for comparison, *n* = 6: *n* = 3 virus-transfected control and *n* = 3 toxin control). Asterisks indicate significance at *p* < 0.01, two-way ANOVA, control versus ablated.

Targeted ablation was achieved by intraperitoneal injection of DT (Azim et al., 2014; Fink et al., 2014). Near complete ablation of GAD67-expressing interneurons can be seen in the immunohistochemical labeling for both GAD67 and GFP in tissue from mice at 6 d after DT (Fig. 3). Colabeling for GAD67 and GFP in the transfected hippocampus confirmed the expression of virus in hippocampal interneurons across all layers of CA1 (Fig. 3*A-C*). At 6 d after DT, in the interneuron-ablated tissue, very few cells expressing GFP or GAD67 could be detected (Fig. 3*D-F*). It should be noted, however, that the ablation did not completely eliminate interneurons from CA1, as some GFP-expressing and GAD67-expressing cells did remain (Fig. 3*G*,*H*). Quantification of the GFP-transfected cells demonstrated a slight bias toward transfection of interneurons in the SO layer, likely due to the injection coordinates. The ablation procedure resulted in a significant reduction of GFP cells in all regions of CA1 (Fig. 3*G*; two-way ANOVA, *F*_(1,16)_ = 85.6, *p* < 0.01 for “treatment: control versus ablated”; *F*_(3,16)_ = 4.2, *p* = 0.02 for “layer”). Likewise, we observed a similarly significant reduction in the number of GAD67 positive cells in the ablated tissue compared to controls (Fig. 3*H*; two-way ANOVA, *F*_(1,28)_ = 43.1, *p* < 0.01 for “treatment: control versus ablated”; *F*_(3,28)_ = 2.9, *p* = 0.05 for “layer”).

### Electrophysiological evidence for selective ablation of interneurons

**Figure 4.**
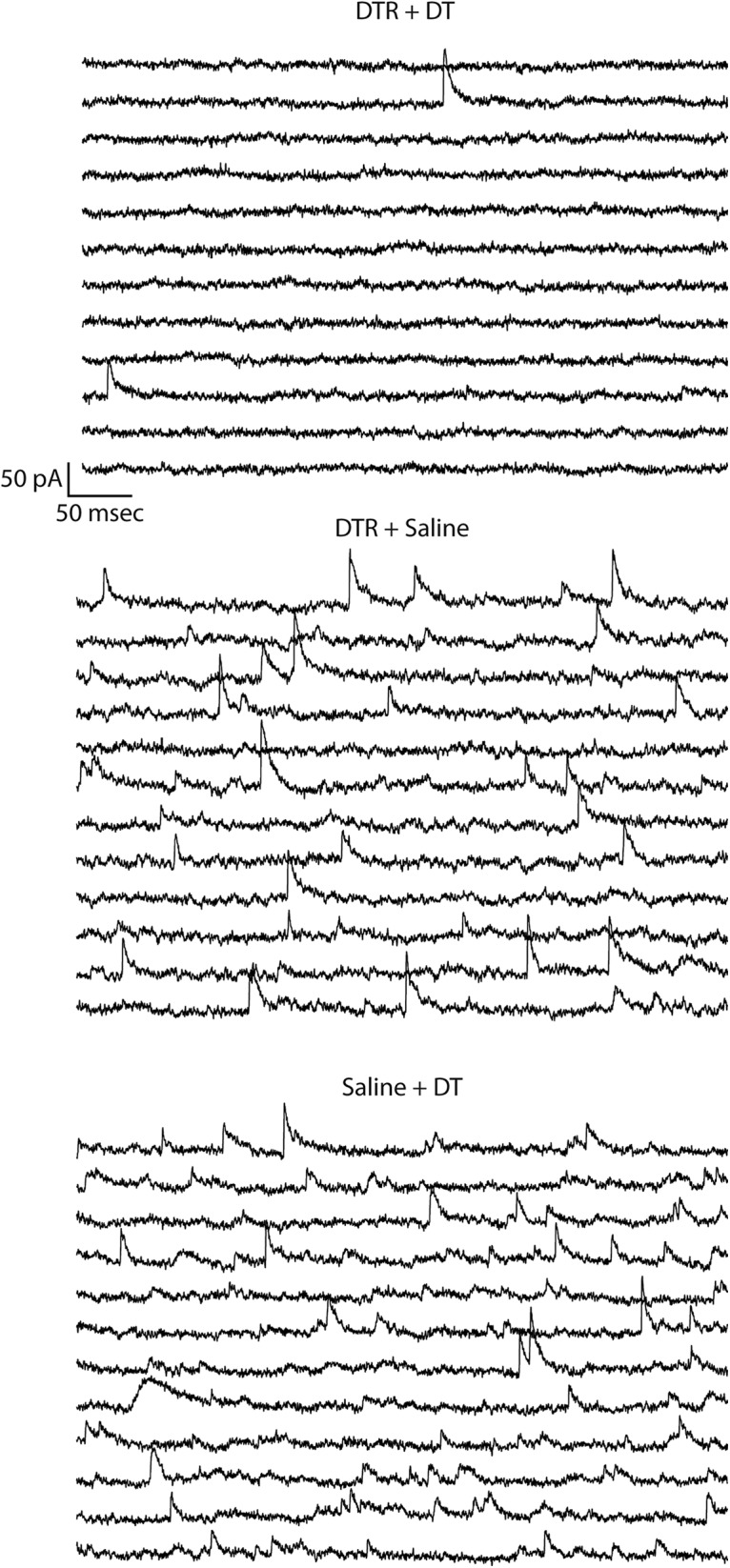
Interneuron ablation results in a specific reduction in the frequency of mIPSCs. Whole-cell recordings from CA1 pyramidal cells demonstrated that the mice that underwent the ablation procedure (DTR + DT) had a reduction in the frequency of mIPSCs. Data are arranged so that the start of each successive line is a continuation of the line above it, and the mIPSCs can be seen as sharp upward deflections from baseline.

**Figure 5.**
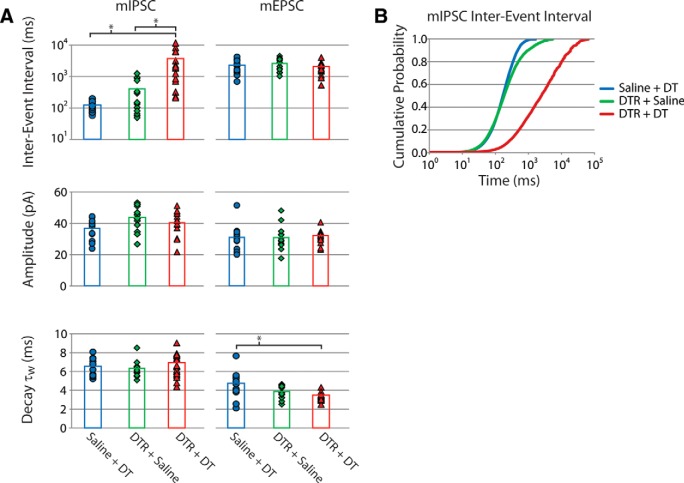
Quantification of the properties of the mIPSCs and mEPSCs. ***A***, The interevent interval, amplitude and decay time of both mIPSCs and mEPSCs recorded in each cell are plotted for saline-transfected controls treated with DT (saline + DT, blue), virus-transfected controls treated with saline (DTR + saline, green) and interneuron-ablated mice transfected with virus and treated with DT (DTR + DT, red). Symbols indicate data from individual cells, and bars indicate the group averages. Data are plotted for the time point of days 6-8 after DT treatment. At this time, CA1 pyramidal cells from the experimental mice had a specific deficit in the frequency of mIPSCs (asterisks, *p* < 0.01, ANOVA; Tables 1, 2) but not the amplitude or decay time, consistent with the loss of inhibitory synapses. ***B***, The >10-fold shift in interevent interval of mIPSCs can also be seen as a large shift in the cumulative probability plot for the DTR + DT compared to controls.

To confirm that targeted interneuron ablation produced an imbalanced network (i.e., that GABAergic synaptic activity was reduced without similar compensatory changes in glutamatergic synaptic activity), we performed whole-cell voltage-clamp recordings from CA1 pyramidal cells. Both mIPSCs and mEPSCs were recorded in the same pyramidal cells from mice that had undergone the interneuron-ablation treatment and from the corresponding control mice, at both 2-4 d and 6-8 d after DT treatment (Figs. 1*B*, 4). The day that the *ex vivo* slice experiment was performed was rotated through the three groups so that each group had a similar number of cells recorded on day 6, day 7 and day 8 after the interneuron-ablation procedure. Likewise, the number of recorded cells/slice/group/d was kept similar so that the results would not be skewed by mismatched sampling. Evidence for loss of GABAergic synapses, or decreased GABAergic synaptic activity, at this time period can be seen in the comparison of the raw data as the interneuron-ablated mice had far fewer synaptic events (Fig. 4). No differences between toxin and virus controls were observed; however, the interneuron-ablated tissue had a >10-fold increase in interevent intervals of the mIPSCs with no changes in amplitude or kinetics (Fig. 5). This effect was specific to the inhibitory synapses, as no compensatory change in frequency or amplitude of mEPSCs was observed. There was, however, a small decrease in the decay time of mEPSCs in the interneuron-ablated mice (Fig. 5*A*). The specific time point of loss of inhibitory synaptic activity occurred after day 4, as there were no significant differences in any of the measured properties between interneuron-ablated and control groups when the *ex vivo* slice experiments were performed at days 2-4 after DT (Table 1, Table 2).

**Table 1.** Synaptic and intrinsic properties of CA1 pyramidal cells at 2-4 d after ablation. Values are given as average (Ave) ± standard deviation (SD).

		**AAV-DTR + DT**	**AAV-DTR alone**	**DT alone**	**Statistics**
		Ave ± SD	Ave ± SD	Ave ± SD	One-Way ANOVA
mEPSC	IEI (ms)	1524.9 ± 933.9	890.0 ± 455.2	1306.5 ± 312.6	*F*_(2,20)_ = 1.76, *p* = 0.20
	Amplitude (pA)	22.9 ± 2.9	21.0 ± 1.1	20.1 ± 3.9	*F*_(2,20)_ = 2.05, *p* = 0.16
	Decay (ms)	4.7 ± 0.9	5.0 ± 0.9	5.3 ± 1.7	*F*_(2,20)_ = 0.40, *p* = 0.67
	*n* (mice/cells)	3/8	3/7	3/5	
mIPSC	IEI (ms)	181.6 ± 154.4	148.2 ± 64.1	124.8 ± 46.0	*F*_(2,22)_ = 0.59, *p* = 0.56
	Amplitude (pA)	35.1 ± 4.6	38.0 ± 5.4	33.5 ± 6.2	*F*_(2,22)_ = 1.36, *p* = 0.28
	Decay (ms)	7.5 ± 1.1	7.5 ± 1.4	6.9 ± 1.4	*F*_(2,22)_ = 0.39, *p* = 0.68
	*n* (mice/cells)	3/8	3/8	3/7	
Intrinsic properties	Whole-cell capacitance (pF)	27.8 ± 5.9	29.9 ± 7.1	27.1 ± 5.6	*F*_(2,22)_ = 0.41, *p* = 0.67
	Input resistance (MΩ)	126 ± 33	129 ± 20	136 ± 39	*F*_(2,22)_ = 0.18, *p* = 0.84
	Membrane time constant (τ_w_, ms)	2.2 ± 0.5	2.2 ± 0.4	2.1 ± 0.4	*F*_(2,22)_ = 0.12, *p* = 0.89

**Table 2.** Synaptic and intrinsic properties of CA1 pyramidal cells at 6-8 d after ablation

		**AAV-DTR + DT**	**AAV-DTR alone**	**DT alone**	**Statistics**
		Ave ± SD	Ave ± SD	Ave ± SD	One-way ANOVA
mEPSC	IEI (ms)	1806.8 ± 805.9	2465.6 ± 1091.5	2199.4 ± 1057.2	*F*_(2,44)_ = 1.75, *p* = 0.19
	Amplitude (pA)	31.7 ± 4.1	32.8 ± 5.9	31.0 ± 7.4	*F*_(2,44)_ = 0.36, *p* = 0.70
	Decay (ms)	3.3 ± 0.4#	3.7 ± 0.6	4.5 ± 1.3	*F*_(2,44)_ = 7.05, *p* < 0.01
	*n* (mice/cells)	4/16	4/15	4/14	
mIPSC	IEI (ms)	3129.0 ± 3391.5*	378.7 ± 433.3	120.5 ± 45.7	*F*_(2,44)_ = 10.3, *p* < 0.01
	Amplitude (pA)	40.1 ± 8.1	43.1 ± 7.3	35.8 ± 6.2	*F*_(2,44)_ = 3.67, p = 0.64
	Decay (ms)	6.8 ± 1.5	6.3 ± 0.8	6.3 ± 0.9	*F*_(2,44)_ = 1.15, *p* = 0.33
	*n* (mice/cells)	4/16	4/15	4/14	
Intrinsic properties	Whole-cell capacitance (pF)	33.6 ± 6.1	30.4 ± 6.7	30.6 ± 6.9	*F*_(2,44)_ = 1.15, *p* = 0.33
	Input resistance (MΩ)	148 ± 68	115 ± 38	100 ± 37	*F*_(2,44)_ = 3.61, *p* = 0.04
	Membrane time constant (τ_w_, ms)	1.9 ± 0.6	2.1 ± 0.6	2.5 ± 0.8	*F*_(2,44)_ = 2.55, *p* = 0.09

* Significant difference between AAV-DTR + DT and both AAV-DTR alone and DT alone (one-way ANOVA, Tukey’s multiple comparison test).

#Significant difference between AAV-DTR + DT and DT alone (one-way ANOVA, Tukey’s multiple comparison test).

### Unilateral dorsal CA1 interneuron ablation caused SRSs

**Figure 6.**
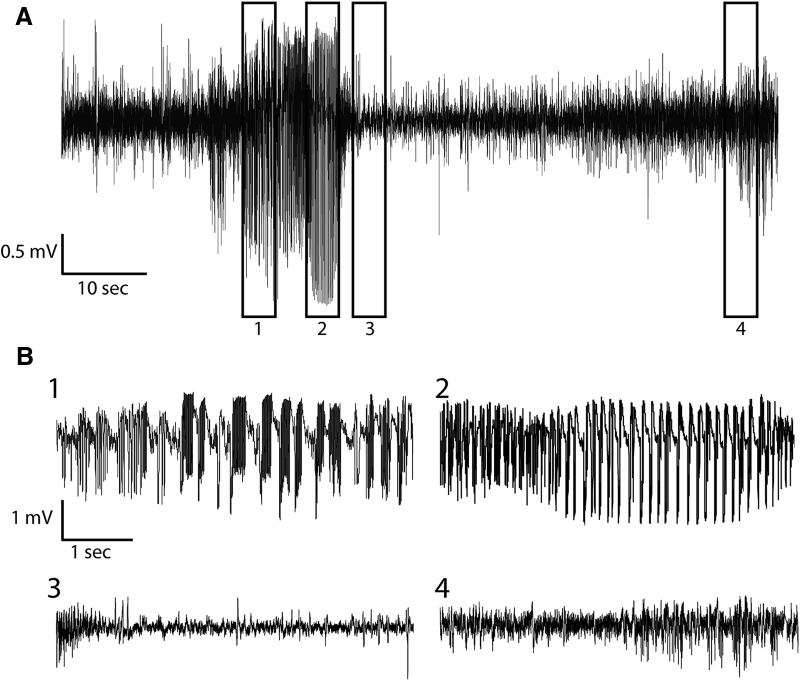
Targeted interneuron ablation produces seizures. ***A***, Example of a seizure recorded in the dorsal CA1 region of the hippocampus using a miniature wireless telemetry recording device mounted to the animal’s head with an electrode positioned near the CA1 pyramidal cell layer. ***B***, Expansion of the boxed regions of the recording in ***A*** demonstrating the changes in the recorded waveforms during the seizure (1 and 2), the postictal depression (3), and return to normal baseline activity (4).

**Figure 7.**
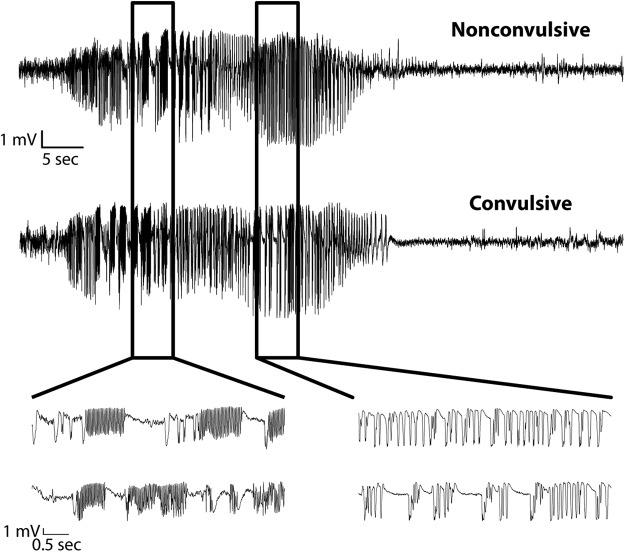
Interneuron ablation produces both convulsive and nonconvulsive seizures. Nonconvulsive and convulsive seizures were indistinguishable in the LFP recordings. The top and bottom seizures occurred ∼8 min apart in the same mouse. The first seizure (top) was associated with behavioral arrest and a gentle head nod side-to-side. The second seizure (bottom) was convulsive with forelimb clonus. Boxed regions are expanded below for both seizures to illustrate the similarities in waveforms during both high-frequency and large-amplitude periods of activity.

**Figure 8.**
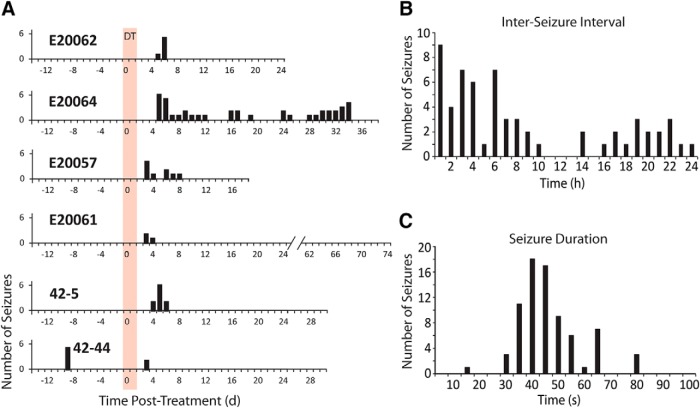
SRSs after interneuron ablation generally occur during a limited time period. ***A***, Daily seizure activity is plotted for two weeks of baseline recording beginning with virus transfection and transmitter implantation. This was followed by continuous recording through the DT treatment period (shown to start at day 0, and lasting for 2 d, red bar) and beyond for several weeks for each mouse. SRSs were seen primarily in the first week after ablation. E20057 had nine seizures spanning from day 3 to day 8; four were confirmed to be convulsive (level 3 or higher). E20061 had three convulsive seizures (two level 3 and one level 5) on days 3-4. E20062 had a total of six seizures over a time period of ∼9 h. Seizures began just before the start of day 6 (11:33 P.M. on day 5); two were convulsive (level 4), four were nonconvulsive (level 2). E20064 had a total of 41 seizures that began on day 5; the first nine seizures were nonconvulsive (level 2), they then progressed to convulsive seizures (level 3-5). 42-5 had 10 seizures on day 4 to day 6; eight were confirmed nonconvulsive (level 2). 42-44 had two seizures on day 3; video from this day was either corrupt or missing. ***B***, Interseizure interval is plotted for all seizure intervals ≤24 h for all mice. The histogram demonstrates that seizures were not confined to brief intervals, thus demonstrating that the interneuron-ablation treatment did not produce a discreet period of SE. ***C***, The histogram of seizure durations illustrates that SRSs induced by interneuron ablation typically had a duration of 30-65 s.

No seizures were found at any time during the combined 445 d of LFP recordings in the control mice. In contrast, all of the mice with the focal interneuron ablation had seizures after DT treatment. Seizures followed a clearly evolving temporal pattern of repetitive high-synchrony and high-amplitude activity with oscillations in different frequency bands, often followed by a period of baseline suppression in the LFP recording (i.e., postictal depression), which eventually recovered back to the normal baseline (Fig. 6). A similar pattern of activity was seen regardless of whether the seizure was convulsive or nonconvulsive (Fig. 7). In all, 47% of the seizures recorded in this study were confirmed to be convulsive by video, while 38% were nonconvulsive; for 14%, the behavioral nature of the seizures could not be determined due to missing or obstructed video. The number of seizures varied from 3-41 per animal (median = 8, mean = 12). In five of the six interneuron-ablated mice, seizures began 3-5 d after DT injection (Fig. 8). The sixth mouse, which also had seizures on the 3^rd^ day after ablation, was observed to have seizures on a single day over 1-week before the ablation treatment. We attribute seizures on this day to an acute effect of the implant surgery, which is not uncommon in humans undergoing an electrode implantation surgical procedure (Coley et al., 2009), and would typically be missed in animal studies that do not use a chronic recording paradigm that begins immediately after implant surgery. The seizures that occurred after the focal interneuron ablation protocol were entirely confined to the first week after ablation for four mice, while one mouse had seizures on the eight day and another mouse continued to have seizures for the length of the recording (Fig. 8*A*). The interseizure interval for seizures occurring ≤24 h apart ranged from <1 h to up to 24 h (Fig. 8*B*), and the seizures lasted on average 45 ± 12 s (Fig. 8*C*; Table 3).

**Table 3.** Seizure properties

**Animal ID**	**Seizure duration (s)**	**Number of seizures**	**Interseizure interval (h:m:s)**		
	Ave ± SD		Ave ± SD	Shortest	Longest
E20057	38 ± 5	9	22:54:26 ± 31:03:27	0:19:35	92:53:34
E20061	29 ± 12	3	3:15:28 ± 3:54:22	0:29:45	6:01:12
E20062	43 ± 3	6	1:53:15 ± 2:17:10	0:07:03	53:15:20
E20064	48 ± 12	41	19:04:33 ± 28:29:36	0:11:29	119:55:47
42-5	61 ± 11	10	8:12:56 ± 6:25:33	2:05:25	19:48:37
42-44	42 ± 8	7	49:51:07 ± 112:08:17	2:49:04	278:44:41
42-44*	38 ± 6	2	5:13:44 ± N/A	5:13:44	5:13:44

* Seizures after the ablation procedure only.

**Figure 9.**
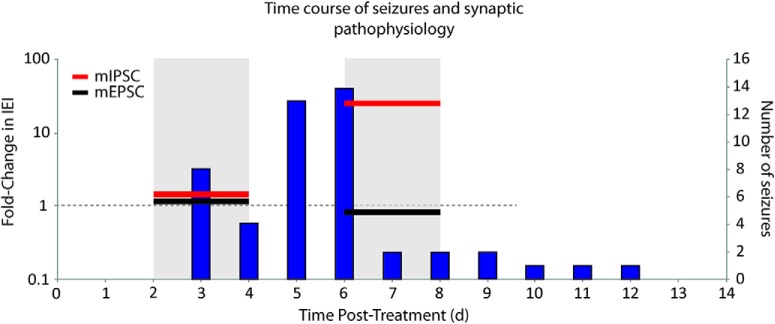
Comparison of the time course of seizures with synaptic pathophysiology. Seizure frequency (i.e., number of seizures per day, right axis) for all mice is plotted (vertical bars) in reference to the fold change in interevent intervals (IEIs, horizontal bars, left axis) of the mIPSCs (red) and mEPSCs (black) recorded from pyramidal cells, in separate cohorts of mice, during the time periods of days 2-4 and days 6-8 after DT treatment. Fold change in IEI was calculated as the ratio of ablated to control so that an increase in fold change indicates a longer interval between events (and therefore lower frequency) in the ablated mice. The occurrence of SRSs is concurrent with the decrease in mIPSC frequency (i.e., the increase in mIPSC IEI). The fold change in IEI of the mEPSCs remains close to 1 at both time periods, demonstrating no concomitant change in excitatory synapses on to pyramidal cells over these time periods.

By considering the time course of seizures in relation to the synaptic deficit identified in the *ex vivo* slice recordings, it appears that the seizures started just before the largest detected deficit of inhibitory synaptic activity. Figure 9 illustrates the time course of seizures combined for all mice in comparison to the observed synaptic physiology presented here. The onset of seizures for the cohort of LFP-recorded mice is concurrent with the loss of miniature inhibitory synaptic activity identified by whole-cell recordings in the separate group of interneuron-ablated mice used for *ex vivo* slice recordings. Interestingly, the halt of seizure activity appears to correlate more temporally with the largest measured imbalance between excitatory synaptic activity and inhibitory synaptic activity between the three groups. It should be noted, however, that these data are purely correlative and causative studies would require knowledge of each individual animal’s seizure history as it relates to the timing of synapse loss, as well as loss of cell function (i.e., precise timing of action potential firing), which was not investigated in this study. In this initial proof-of-principle study, we can only conclude at this time that a targeted interneuron loss in the dorsal CA1 area of the hippocampus is sufficient to produce an imbalance of synaptic activity and SRSs.

## Discussion

We have demonstrated that unilateral ablation of a heterogeneous population of interneurons, localized to the dorsal CA1 region of the hippocampus, is sufficient to produce nonconvulsive and convulsive seizures. We aimed to determine if these seizures had the properties of SE and/or chronic epilepsy. Both nonconvulsive and convulsive seizures were tens of seconds in duration with interseizure intervals of several hours (or even days), which indicated that DT-mediated interneuron ablation did not induce SE; instead, the pattern of SRSs had similarities to chronic epilepsy. The SRSs were first observed at 3-5 d after DT treatment, which, based on prior work, was the expected time required for DT-induced death of interneurons (Azim et al., 2014; Fink et al., 2014). Immunohistochemistry of GABAergic interneurons and whole-cell recordings of mIPSCs from CA1 pyramidal cells supported the hypothesis that the first SRSs occurred as the interneurons were dying and GABA_A_-mediated inhibition was sufficiently depressed; therefore, the 3-5 d delay between DT administration and the occurrence of the first SRS was almost certainly due to the time required for DT to kill the interneurons, thus indicating that the SRSs occurred without the latent period typical of acquired epilepsy. The SRSs stopped within several days for 5 out of 6 DT-treated mice, thus suggesting that the DT-induced interneuron lesions caused SRSs, but their failure to continue beyond a few days indicates that the interneuron ablation did not typically cause *bona fide* epilepsy. In one instance, however, seizures occurred for ≥34 d after interneuron ablation, similar to epilepsy after experimental SE. Sham control mice had no detectable seizures, confirming that the SRSs were due to ablation of interneurons. In conclusion, these data show that selective interneuron ablation consistently caused SRSs that did not have the properties of SE; however, at least under the conditions used here, interneuron lesions rarely led to persistent SRSs (i.e., epilepsy). These data establish the usefulness of this interneuron-ablation technique for the investigation of the role of interneurons in abnormal circuit function in cortical structures of freely behaving mice.

### Time course and magnitude of interneuron loss

#### Time course

DT kills cells by inhibiting amino acid polymerization at the ribosome, thereby eliminating protein synthesis (Collier, 1975). This irreversible cessation of protein synthesis leads to severe metabolic stress and subsequent cell death. Fink et al. (2014) previously demonstrated a timeline for the killing of neurons in the spinal cord of mice via this same DT receptor-mediated ablation technique, and they reported neuronal death to occur within 4-6 d after DT treatment, based on measurement of a behavioral deficit. We have observed a similar time course for the loss of mIPSC activity in hippocampal CA1 pyramidal cells, which appeared maximal by day 6 after DT administration. Our data suggest that seizures began to occur during DT-induced interneuron death, possibly at the initiation of cell death.

#### Magnitude

This interneuron-ablation technique killed ∼88% of the countable GAD67-labeled interneurons within the estimated lesional area, leaving ∼12% of the countable interneurons still alive at 6 d after DT. Immunohistochemistry revealed that some of these interneurons were not transfected by the virus (i.e., no expression of GFP), and some transfected interneurons (GFP expressing) were not ablated by the toxin (Fig. 3). Electrophysiological assessment of inhibitory synaptic connectivity demonstrated a >10-fold decrease in the frequency of mIPSCs by day 6-8 after DT administration. It is not clear how a decrease in the frequency of mIPSCs relates to the proportion of GABAergic interneurons no longer providing input to the CA1 pyramidal cells, but the dramatic decrease in mIPSC frequency combined with the immunohistochemical data converge on the conclusion that most GABAergic interneurons were killed in the area of the lesion. Whether the remaining synaptic activity was from interneurons within or outside the ablation area is unknown. In the context of focal epilepsy, this interneuron ablation technique created a large lesion (diameter estimated to be ≥1200 μm) along the septo-temporal axis of the hippocampus with a nearly complete elimination of countable GABAergic interneurons. Thus, the failure to consistently induce intense SE or chronic epilepsy with persistent SRSs was not because the lesion was small or ineffectual, but an even larger lesion or interneuron ablation in other areas, such as the ventral hippocampus, might be more efficacious for induction of epilepsy (Ekstrand et al., 2011).

The most detailed and rigorous analysis of interneuron loss associated with the DT-mediated ablation procedure used here would have entailed a volumetric determination of the actual number of neurons in reference to the three-dimensional coordinates of the dorsal CA1 region of the hippocampus. For this proof-of-principle study, however, we only aimed to determine the relative magnitude of the interneuron loss in the experimental and control groups across the entire volume of tissue studied. Thus, we did not attempt to use stereological techniques, which would be the traditional approach for quantitative analyses of the three-dimensional nature of the interneuron loss. Scanning confocal microscopic analysis of “cleared” blocks of tissue would provide an even more direct method for obtaining quantitative information on the three-dimensional shape of the lesion area. Future studies aimed at more specific questions concerning the effects of interneuron lesions would benefit from these much more difficult but rigorous methods.

The analysis of interneuron loss from the DT-mediated ablation procedure presented here only provides a relative comparison based on a limited number of sections through comparable areas of the dorsal CA1 from the two groups of animals. Due to incomplete penetrance of our fluorescence probe (anti-GAD67), we were unable to count cells through the entire *z*-axis of each section, and therefore, we limited our counting to the surface of each section. Our protocol incorporated the visual observation of the complete cell body for inclusion of an interneuron in the tally (i.e., we excluded cell fragments), so we may have underestimated the number of interneurons in the samples; however, this potential problem is unlikely to have biased the results, because the potential underestimation would be similar for both the experimental and control groups. Our analysis of the anatomic data shows that the DT-mediated ablative procedure reliably caused a specific and relatively consistent interneuron lesion, and that most of the interneurons at the injection site were killed.

### Interneuron death is sufficient to cause SRSs

Toxin-mediated, selective removal of interneurons from the hippocampus has previously been attempted using saporin targeted to interneurons via ligand conjugation to allow binding and internalization through either the substance P receptor or the vesicular GABA transporter (Martin and Sloviter, 2001; Zipancic et al., 2010; Antonucci et al., 2012). These studies demonstrated that interneuron loss can produce a hyperexcitable network, but neither study demonstrated SRSs, possibly because of limited *in vivo* monitoring. Furthermore, these previous interneuron-ablation techniques produced either gross morphologic changes in hippocampal anatomy or interneuron loss beyond CA1 (Martin and Sloviter, 2001; Zipancic et al., 2010; Antonucci et al., 2012). In our study, continuous chronic monitoring of hippocampal LFPs overcame previous concerns about a relatively low occurrence of seizures and the presence of nonconvulsive seizures. SRSs were observed here in every mouse subjected to the interneuron ablation technique, and this was a result of the specific ablation of a discreet population of interneurons confined to the CA1 region, with no other gross morphologic or anatomic changes in the hippocampus (data not shown). Our data therefore demonstrate that focal interneuron loss alone is sufficient to produce SRSs.

### Lack of occurrence of SE

Although the DT-mediated interneuron-ablation procedure caused SRSs at approximately the time when interneurons would be expected to be killed, and although the lesion was large (i.e., ≥1200 μm) and extensive (i.e., within the limitations of our assessment, most interneurons in the lesional area were killed), SE was never observed. The classical definition of SE can be summarized as the occurrence of a prolonged seizure >30 min or repetitive seizures occurring for a period of >30 min. In the present experiments, the individual SRSs were relatively brief in the context of SE, with SRS durations of 30-80 s and SRSs being separated by long interseizure intervals that were typically >1 h; thus, the properties and patterns of SRSs were quite different from what is generally observed during SE (Lehmkuhle et al., 2009; Phelan et al., 2015). However, in terms of the duration of the seizures and the interseizure intervals, the SRSs observed here after interneuron ablation appeared quite similar to the SRSs described for several different animal models of acquired epilepsy (e.g., Statler et al., 2009; Williams et al., 2009; Kadam et al., 2010). The fact that five of six mice stopped having SRSs after only a few days strongly suggested that the SRSs were primarily an expression of the acute/subacute occurrence of seizures and were not tantamount to chronic epilepsy. This observation suggests that the SRSs observed here were more similar to those observed in the first few days after an epileptogenic insult (i.e., the “early” seizures) and were not truly representative of chronic epilepsy (i.e., with “late” seizures).

### Does interneuron death cause epilepsy?

An important hypothesis on the cellular and circuit changes that occur during epileptogenesis suggests that neuronal death, including interneurons, is a necessary, or at least contributory, component; however, synaptic reorganization may also be required or at least important for the establishment of an epileptic network (Sayin et al., 2003; Cossart et al., 2005; Maglóczky and Freund, 2005; Dudek and Sutula, 2007; Sutula and Dudek, 2007; Dudek and Staley, 2012). In the slow epileptogenesis modeled by kindling, selective interneuron loss and reduction of inhibition have been reported to correlate with the emergence of SRSs when compared to animals that have undergone significant kindling but have not developed SRSs (Sayin et al., 2003). That is, despite the extensive kindling-associated physiologic changes (for review, see Morimoto et al., 2004), the presence of selective interneuron loss was most directly associated with development of epilepsy. Our experiments aimed to directly test the hypothesis that interneuron death alone is sufficient to produce epilepsy.

Epilepsy is clinically defined in humans as two or more unprovoked seizures occurring >24 h apart. Using this definition, each of the mice with an interneuron lesion in the present study could be considered to have acquired epilepsy, because each mouse experienced two or more SRSs. However, with the exception of one mouse, the SRSs underwent remission. That is, the SRSs did not continue beyond one week, although the interneurons were permanently ablated. Interneuron ablation could potentially be considered a form of traumatic brain injury, in which case it could be argued that these seizures occurred in the acute or subacute injury-associated period, and this type of injury may not necessarily be expected to result in epilepsy. If the immediate time period of the ablation is assumed to represent an acute injury, only one animal developed epilepsy with chronic SRSs. The transient, nonprogressive nature of the seizure phenotype observed here, however, suggests that a compensatory mechanism was activated to suppress SRSs (and epileptogenesis), as has been seen in neurons that have lost tonic GABA conductance through the genetic knock-out of extrasynaptic receptors (Brickley et al., 2001). Given that the dorsal CA1 is classically thought not to be as epileptogenic as the ventral hippocampus or amygdala, it remains possible in future experiments that an interneuron lesion in one of these other areas could cause the mice to have a progressive and permanent epilepsy (Racine et al., 1977; Gilbert et al., 1985; Akaike et al., 2001; Ekstrand et al., 2011; Scholl et al., 2013; Toyoda et al., 2013).

### Is there a latent period?

In regard to a possible latent period for acquired epileptogenesis, the simplest and most parsimonious interpretation of our data are that unilateral CA1-targeted interneuron ablation resulted in a period of SRSs without a latent period (for review, see Dudek and Staley, 2011, 2012; Löscher et al., 2015). Virtually all of the time between the ablative treatment and the first SRS (3-5 d) could be accounted for by the known cellular mechanism of DT-mediated neuronal death. One alternative interpretation of our results, however, is that the DT-induced interneuron death was followed by an acute injury-induced period of “provoked” seizures (i.e., in four of the six mice, the SRSs only occurred up to 6 d after the first injection of DT). In this interpretation of the data, the proposed “remission” of seizures may have been the actual latent period for subsequent SRSs that might have been detected if the recordings were longer. In the 5 mice where the SRSs appeared to undergo remission, the time from the last identified seizure until the end of the recordings ranged from 10-70 d. As our mice did not experience injury- or DT-induced SE, but instead began to have SRSs with interseizure intervals and seizure frequencies more typical of an established epilepsy phenotype, we find it more likely that the interneuron-ablated mice developed SRSs with virtually no latent period. However, the duration of the latent period can vary dramatically depending on the nature and severity of the injury/induction method (i.e., chemoconvulsant, hypoxia-ischemia, traumatic brain injury, etc.). It, therefore, remains possible that the interneuron ablation performed in this study induces a form of epileptogenesis with a long delay from the initial injury and its injury-induced acute seizures (i.e., during the first week after DT injection) to the chronic SRSs typical of established epilepsy (Curia et al., 2008; Williams et al., 2009; Kadam et al., 2010; Dudek and Staley, 2012; Lévesque and Avoli, 2013; Löscher et al., 2015). Longer recordings will be necessary to determine if the SRSs were temporarily or permanently suppressed after the first week, and to assess mechanisms of the associated latent period or remission, respectively. Likewise, a larger group size will be necessary to determine the true rate of development of SRSs that persist beyond the first week.

## Conclusions

This study was intended as a proof-of-principle and should be considered as such in regards to the low group size for immunohistochemistry and chronic EEG monitoring. We used a minimum number of animals to test the core hypothesis that interneuron loss can cause seizures. We have found that a discreet interneuron-selective hippocampal lesion in an otherwise normal brain is sufficient to produce SRSs in six out of six animals tested. However, these SRSs usually entered a prolonged period of remission within the first week after the ablation procedure (five out of six animals). These data suggest interneuron loss supports the development of epilepsy, but alone, the interneuron loss induced in these studies was not a cause of progressive and prolonged epilepsy. This is consistent with a “two-hit” hypothesis that chronic, selective interneuron ablation with a transient period of SRSs requires additional cellular or network alterations to result in acquired epilepsy. Subsequent studies with larger group sizes, however, will be necessary to determine the specific role of interneuron loss in acquired epilepsy. This new model should also be useful in determining the role of different interneuron subtypes in the generation and remission of SRSs.

## Synthesis

Reviewing Editor: Maiken Nedergaard, University of Rochester Medical Center Decisions are customarily a result of the Reviewing Editor and the peer reviewers coming together and discussing their recommendations until a consensus is reached. When revisions are invited, a fact-based synthesis statement explaining their decision and outlining what is needed to prepare a revision will be listed below. The following reviewer(s) agreed to reveal their identity: Thomas Sutula

Both reviewers appreciated the study, but have many suggestions for how to improve the manuscript. We are looking forward to receiving a revised manuscript.

Reviewer #1

In this study, Spampanato and Dudek show that ablating GABAergic interneurons in dorsal CA1 is sufficient to induce spontaneous recurrent seizures, which normally show remission within one week. There are several aspects of this process that could be examined in more detail, but the current proof-of-principle study has been performed carefully, and appears suitable for rapid publication in an open access journal.

I only have minor comments:

1) There is the potential for changes in intrinsic excitability following loss of GABAergic inhibition (for example, Brickley et al, 2001, doi:10.1038/35051086), which could contribute to seizure remission. It may be beyond the scope of this study to explore this in detail, but it would be useful for the authors to report the whole-cell capacitance and input resistance measurements from their voltage-clamp recordings. This could be included in Tables 1 & 2.

2) As stated in line 242, all but one of the mice undergoing video-LFP recordings survived until the battery on the recording device died. Were the mice perfused for analysis of electrode placement and lesion size? The design presented in Fig1 suggests this is not the case - if so, could the authors explain the rationale and/or technical reasons for this.

This information might be particularly informative in understanding the seizure profile in mouse E20064, which appears to have the most early seizures (within 1 week post DT), as well as persistent seizures. Could this simply be explained by the size / extrahippocampal spread of the lesion?

3) Statistical reporting - the authors should report F and t statistics, degrees of freedom, and precise p values. For analysis of the electrophysiology data, this information could be included in Tables 1 & 2.

4) Lines 179 & 182 - appears to be error in formula for sodium bicarbonate

5) Line 399 - ‘saponin’ should be ‘saporin’

6) Fig3 Currently panel F labelled as ‘Post-DT Ipsilateral CA1’. The layout should be altered to make it clear that this label applies to panels D-F, and provide a similar ‘Virus control’ label for panels A-C.

Panels G & H - seems more appropriate to use a mixed measures ANOVA design here, to explore the main effect of treatment, and potential interaction between treatment x layer

7) Fig 5 & Table 2 - asterisks are reported to denote significance with ANOVA. This must be result of posthoc pairwise comparisons, and the full details of the tests used need to be described.

Reviewer #2

This is a clearly designed, carefully executed, and nicely presented study combining lesioning methods, histology, and both in vitro and in vivo electrophysiology to investigate classic questions about epileptogenesis and role of inhibition/interneurons. The technical strength of the study is the application of contemporary methods enabling highly selective targeting of a subset of interneurons with precision previously impossible using older lesioning methods. The clarity of the lesioning enabled rigorous temporal analysis of the onset of cellular abnormalities of IPSCs and the correspondence to in vivo seizures. The most interesting aspect of the results was unanticipated, namely that status epilepticus was not induced and brief periods of nicely documented spontaneously recurring seizures a short interval did not, except for 1 animal, lead to recurring chronic seizures that define epilepsy. The following comments are intended merely as points of clarification that could further increase interest in this nicely performed and presented study.

In the Methods on p. 7, it appears cell profiles were counted in the absence of formal stereological methods. More detail about counting methods and its limitations in the absence of stereological assessment would improve the presentation of the results and appreciation of the strength of the observations and comments. More critical statements about this point and what would or would not be gained by the effort of a formal stereological analysis would strengthen the comments and interpretation in the Results and Discussion.

On p. 9,the relatively small number of subjects but probably OK. This point would benefit from explicit consideration, probably best in the discussion.

In the Results, on p. 10, the statement about a high density of cells may be a bit judgmental - punctae certainly prominent, cells fewer. This observation is likely to consistent with widely ramifying interneuron projections. This could be clarified by ultrastructural or stereological methods, again deserving explicit comment that would strengthen the reasonable interpretation.

In the Discussion on P. 14, the statement ‘This interneuron-ablation technique appeared to kill nearly all of the interneurons within the center of the lesional area’ may be too strong in the absence of stereological assessment.

On p. 15 of the Discussion, the authors state ‘these previous interneuron-ablation techniques produced either gross morphological changes in hippocampal anatomy or interneuron loss beyond CA1.’ This is a substantial point relative to previous methods and work that deserves more emphasis. Pertinent to this significant point, the authors may wish to cite a previous publication in the kindling model demonstrating that selective interneuron loss and functional loss of inhibition correlate with emergence of spontaneous seizures in kindled rats (Sayin et al J Neurosci, 2004). These observations in the slow epileptogenesis process modeled by kindling with a slow process of neuronal loss and other circuit alterations support the conclusions of the this paper that more than interneuron loss is required to establish epilepsy, and could strengthen the perspective the authors are seeking to emphasize.

On p. 15, the authors might want to mention the possibility that epilepsy could still develop at longer intervals after the induced interneuron loss - this has not been excluded. There still could be a latent period process, just a longer one given the relatively selective lesion and the possibility that eventually other events required for SRS could ‘catch up’.

On p. 16, the human definition of epilepsy is 2 seizures more than 24 hours apart. This is still an arbitrary interval, and it could be argued that seizures over several days due to a specific insult such as focal interneuron loss/pathology will not inevitably lead to epilepsy. Strict comparison to the also arbitrary human definition could argue that these mice had epilepsy that remitted, which is obviously a semantic point that would benefit from further consideration and clarification.

The interesting unanticipated aspect of the study indicating the complexity of alterations in neural circuitry beyond interneuron loss probably deserves more emphasis in the discussion, and possibly in the abstract.

The authors analysis and comments about the extent of the interneuron ablation probably deserve a bit more critical emphasis. Stereological measurements would further strengthen their interpretation but are probably not necessary, but more acknowledgment of the limitations based on a formal quantitative stereological analysis would add critical credibility.
